# A Dentist in the Newspapers

**Published:** 1875-06

**Authors:** 


### ARTICLE V.

### *A Dentist in the Newspapers.*

A dentist of Washington, D. C., has been attracting some rather unenviable attention from the newspapers of that city, by the publication of a very singular and mysterious card, as follows:

"WANTED—The front teeth of a girl fourteen years of age. Will pay liberally and replace artificially. Apply after 3 P. M., at Dr. ——."

This advertisement very naturally called forth the wondering comments of many who read it. Why the teeth of a boy of that age wouldn't do, and why, indeed, teeth at this exact time of life. It does not appear, however, very clearly from the card, whether the patient or the teeth should be fourteen that were wanted.

If our dentist did not get the desired "front teeth," he seems at least to have gotten notoriety, as appears from the following card, published soon after the appearance of the advertisement in question.

THAT FRONT-TOOTH ADVERTISEMENT.

*To the Editor of the National Republican.*—SIR: A dentist offers to pay liberally for a front tooth of a girl fourteen years of age, and to replace artificially. Upon the commission of this act will not the offender, on conviction, be imprisoned not exceeding seven years and fined not exceeding $1,000 under the act of Congress of the 30th of April, 1790, section 3. To extract a sound front tooth is such a bodily hurt as not only maims and disfigures, but diminishes corporal abilities. Anciently it was held to be felony, because the offender had judgment of the loss of the same member. The words of the statute are very full and comprehensive, and extend to deforming a poor female child by pulling out a fore-tooth for mercenary purposes. The press, the public censor, should rebuke such an insult to the morals and humanity of the age. Where is the Society for the Prevention of Cruelty, not to Animals, but to Children. K.

And not content with what his correspondent says in the matter, the editor of the paper adds the following:

The attention of Mr. Bergh, Mr. Gatchell and the society generally for the Prevention of Cruelty to Animals, is directed to a daily advertisement in an evening cotemporary, as follows: "Wanted.—The front tooth of a girl fourteen years of age. Will pay liberally and replace artificially." The front *teeth* were first called for; the number is now modestly reduced to one. In the imaginative pages of Eugene Sue the agonies of his lovely and unfortunate heroine, "Fleur de Marie," are graphically portrayed while undergoing a like torture; and Victor Hugo, too, in the career of the still more wretched "Fantine,' presents us with a similar episode. These are fictions only—the wild exaggerations of French romance—but for our happy and enlightened country it is reserved to be the proposed theatre of the actual, horrible *fact*, provided only the victim stands ready. Gentlemen of the humane society, do you consent? Read the communication in another column regarding this matter.

These criticisms on his call for teeth brought out the dentist himself, in this style:

WASHINGTON, D. C., Feb. 5, 1875.

*To the Editor of the National Republican.*—SIR: Desiring to solve a great scientific problem, I have become the subject of severe criticism, though no injustice or inhumanity is contemplated or necessary. Many teeth are sacrificed from various causes that will answer my purpose; it is not even necessary that they should be sound. The principle I believe to be correct. If it proves so, the poor will have reason to consider me a benefactor.

Signed, ——— ——— ———, D. D. S.

This card it appears was put forth to silence clamors of the sensitive public, who were fearing the heartless mutilation of some needy creature, who would be willing to sell her teeth (why not *his* teeth?) for cash. But the card itself appears a greater enigma than the publication which had produced the clamor. What "great scientific problem" could be possibly solved by the loss of a few teeth by a poor girl, one is at a loss to see.

And what principle could be established as "correct" it would have been well if this D. D. S. had stated. He, however, "believes it to be correct," and if he proves it to be so

"the poor will have reason to consider him a benefactor." Benefactors of the poor are not so numerous as they might be, and it is refreshing to know that a process is to be de rived by which this benefaction can be brought about by the loss of a tooth or teeth. Many a dentist and for that matter, a country blacksmith, has been called blessed by those he had relieved of the tooth-ache by sundry violent wrenchings at an offending molar, but here is a man who it seems can only be a benefactor through the agency of the front teeth of a girl of fourteen ; to whom he "contemplates no injustice or inhumanity;" and they had not been sound, he adds:

Many people lose their teeth, some from neglect, many it is found by the incompetency of dentists, many from the inherent tendency of all things to go to work and ruin,—except principles. And so our D. D. S., knowing this, and wishing to establish the correctness of a "principle," wants "the" front teeth of a girl fourteen years old; "apply after 3 P. M."

It would have been vastly better for our D. D. S., better for his reputation as a practitioner and as a means of escape from his rather merciless critics—perhaps from the "Society for the prevention of cruelty to animals," if he had written either nothing at all, or else said plainly what is plain to every professional man interested in the case, that he wished to renew the old and long disused practice of transplantation, practised in the last century, and caricatured by Roulordson, a famous artist of his day, a wood cut of which caricature has been lately published in a contemporary Journal; an operation so unfeeling and barbarous as to cause wonder at its ever having been tolerated. That this was the object contemplated cannot admit of a doubt,—no other could be assigned, but, that in this day of enlightenment a practitioner—a D. D. S. at that,—should be so behind the times, so little posted as to the valuelessness of such an operation in view of its great uncertainty is strange.

It is considered by some as an operation of sufficient safety to warrant its occasional performance to extract an abscessed tooth that has defied other treatment, and replace after proper treatment; and even this the returning of a tooth to its own well filling socket is considered as not at all reliable; but that any one in this day should seriously contemplate a revival of the obsolete and cruel operation of transplantation, the mutilation of one poor girls' mouths for the temporary adornment of some one with money, but weak and foolish enough to allow this sort of experimentation is a little startling for this day of dental enlightenment.

In the caricature referred to, two figures are represented as going out of the door, with the money received for their sold incisors, apparently well satisfied.

These are the poor who were "benefacted" by the dentist of that day. Is it in this way that our D. D S. proposes to make himself the peer of the Corcoran's and Smithsons', who have conferred on the poor of Washington so many real blessings. Verily, to lose one's front teeth and pay for their extraction is not counted a benefit by any means, and that a remuneration to the amount of even fifty dollars would be considered an equivalent for this loss is hardly to be believed. A later card shows that the Dr. has not given up his idea of getting a front tooth, and he now, it seems does not specify sex. This card is headed

PERSONAL.

$50 is the value that will be paid for the front tooth of a young person, even (though paining and not sound) if answering the Doctor's purpose.

---

